# The Prevalence of *Klebsiella* spp. Associated With Bovine Mastitis in China and Its Antimicrobial Resistance Rate: A Meta-Analysis

**DOI:** 10.3389/fvets.2022.757504

**Published:** 2022-06-24

**Authors:** Kai Liu, Limei Zhang, Xiaolong Gu, Weijie Qu

**Affiliations:** College of Veterinary Medicine, Yunnan Agricultural University, Kunming, China

**Keywords:** bovine mastitis, *Klebsiella* spp., epidemiology, antimicrobial resistance, meta-analysis

## Abstract

Understanding distribution of bovine mastitis pathogen *Klebsiella* spp. can contribute to the treatment decision and the control within programs of bovine mastitis, we conducted a meta-analysis to investigate the epidemiology and antimicrobial resistance rates of *Klebsiella* spp. associated with bovine mastitis in China. Three databases, namely, PubMed, Google scholar, and China National Knowledge Infrastructure database, were utilized to obtain relevant publications. According to PRISMA reporting standards, a total of 38 publications were included in the research, among them, 7 papers included an AMR test. The pooled prevalence of *Klebsiella* spp. was 5.41% (95% CI: 3.87–7.50%). Subgroup analysis revealed that the prevalence was higher in South China (8.55%, 95% CI: 3.57–19.09%) than in North China (4.22%, 95% CI: 2.46–7.14%), in 2010–2020 (7.45%, 95% CI: 5.29–110.40%) than in 2000–2010 (3.14%, 95% CI: 1.90–15.14%), and in the clinical bovine mastitis cases (7.49%, 95% CI: 3.71–14.54%) than in the subclinical cases (4.03%, 95% CI: 1.55–10.08%). The pooled AMR rate revealed that *Klebsiella* spp. were most resistant to sulfonamides (45.07%, 95% CI: 27.72–63.71%), followed by tetracyclines (36.18%, 95% CI: 23.36–51.34%), aminoglycosides (27.47%, 95% CI: 17.16–40.92%), β-lactams (27.35%, 95% CI: 16.90–41.05%), amphenicol (26.82%, 95% CI: 14.17–44.87%), lincosamides (21.24%, 95% CI: 7.65–46.75%), macrolides (20.98%, 95% CI: 7.20–47.58%), polypeptides (15.51%, 95% CI: 6.46–32.78%), and quinolones (7.8%, 95% CI: 3.25–17.56%). The climate difference between South and North China and the natural pathogenicity of *Klebsiella* spp. may be the primary reasons for its distribution, and the prevalence of *Klebsiella* spp. indicated that the genus is an increasing hazard to the dairy industry. The prevalence of AMR in China is commonly higher than in the European countries and Canada, this is a very important concern for strategy programs to control bovine mastitis caused by *Klebsiella* spp. in China.

## Introduction

Mastitis is one of the costliest diseases in the dairy industry due to the discarding of milk and expenses of treatments, including the culling of cows (1–3). *Klebsiella* spp. are the major gram-negative pathogens that cause mastitis (4–7), and the concern for their pernicHiousness to the dairy industry in China has increased in recent years (8). In recent research, *Klebsiella* spp. was isolated from 13% of clinical bovine mastitis samples collected from dairy farms in China (9). *Klebsiella* spp. mastitis is prolonged with a severe and long-lasting duration of intramammary infection and is often accompanied by a considerable decrease in milk production (10); this condition shows no desirable response to antimicrobial treatments (5, 11). Consequently, cows with *Klebsiella* spp. mastitis are more likely to be culled compared with cows with other types of mastitis (12–15).

Antimicrobials are still the major option for the treatment of mastitis (16). However, the abuse of antimicrobials increases the risks of antimicrobial resistance (AMR) in bacteria, which is a worldwide public health concern (17, 18). Alvarez-Uria et al. predicted that a considerable proportion of *Klebsiella pneumoniae* will likely be resistant to carbapenems and third-generation cephalosporin in most parts of the world by 2030 (19). The “National action plan to combat animal resources antimicrobial resistance (2017–2020). Beijing: China Ministry of Agriculture and Rural Affairs; 2017,” is one of the national protocols for standardizing veterinary medication, along with strict biosecurity, sterile standard, and the prudent use of antimicrobials to release the pressure of transmission of antimicrobial-resistant pathogens. Wang et al. (20) reported that the policy and decreased use of colistin in agriculture had a significant effect on the reduction of colistin resistance in animals and humans in China.

Investigation of the epidemiology and AMR profiles of *Klebsiella* spp. can contribute to treatment decisions and optimization of *Klebsiella* spp. control programs (21). Numerous publications focused on the AMR of other major bovine mastitis pathogens in China, including *S. aureus* and *E. coli* (22, 23), whereas the meta-analysis can overcome the insufficient spatial and temporal distribution of *Klebsiella* spp.

## Materials and Methods

### Literature Search

**Figure 1:**
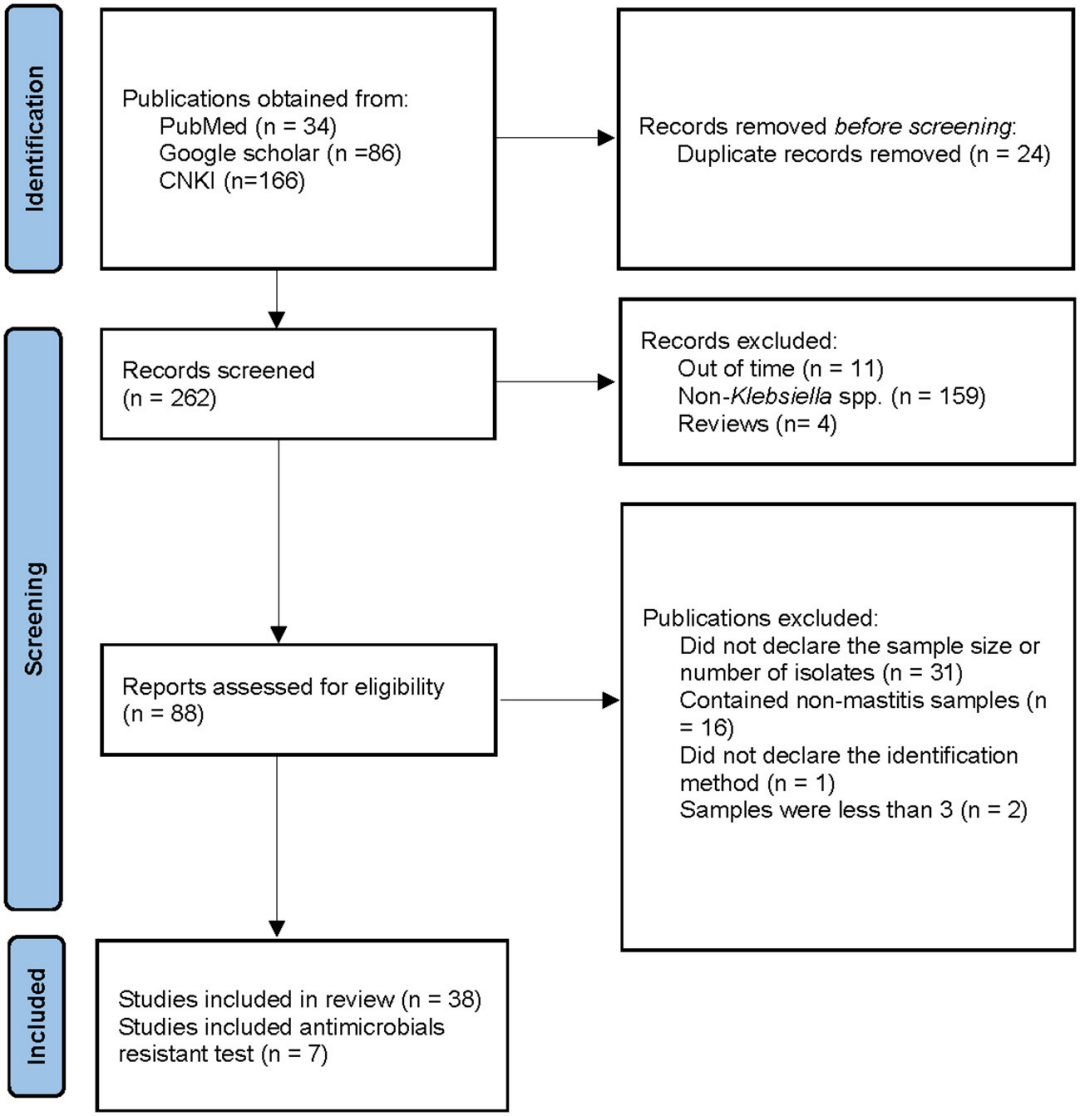
Search and selection criteria for literatures. CNKI means Chinese National Knowledge Infrastructure.

Figure 1 illustrates the relevant steps and results of the literature retrieval. For a previously published review, a comprehensive and systematic literature search was conducted by two independent reviewers on 23 May 2021, utilizing the PubMed (http:// www.pubmed.gov), Google scholar (https://scholar.google.com), and China National Knowledge Infrastructure (CNKI) databases (https://www.cnki.net/) to identify the literature focusing on Klebsiella mastitis in cows. The subject heading “bovine mastitis AND bacteria” was used to find all trials on this topic written in the English or Chinese language. The time was set from 2000 to 2021 to assure the timeliness of the subsequent meta-analytic investigation.

### Inclusion and Exclusion Criteria

As reported previously (24), our study was in accordance with PRISMA reporting standards (25), specific exclusion criteria were defined to exclude articles that did not describe clinical trials (e.g., descriptive, or *in vitro* studies). Two authors reviewed all abstracts and then performed a full-text review of articles for eligibility independently, the agreement between the two reviewers for inclusion of articles was good (κ = 0·86). The excluded publications included review articles, articles did not meet the inclusion criteria due to wrong indexation (“off topic”), out of the considered time period, small sample size (less than three samples), exclusion of Klebsiella, undeclared bacterial identification method, samples containing non-mastitis diseases, undeclared sample size or number of bacterial isolates, and unobtainable through the internet. Then, the two reviewers extracted the data from included articles independently. Retrieval and management of references were performed with Excel (Office 16 for Windows, Microsoft Office, New York, USA) (Table 1).

**Table 1:** Information of literatures included in our study.

**Author**	**Publication year**	**Samples**	**Identification assay^**1**^**	**Case**	**Grade^**2**^**	**Region^**3**^**	**AMR method^**4**^**
Linzheng Jiang	2020	31	16S	2	–	S	K–B
Lan Liu	2009	32	other	9	–	NS	–
Ling Wang	2020	37	16S	12	C	S	–
Xiujuan Ye	2004	44	other	3	–	S	–
Ridong Guo	2015	45	other	3	C	NS	–
Qiuyun Zhao	2016	48	other	1	C	NS	–
Mingxu Zhou	2019	50	16S	13	S	S	K–B
Chengyi Zhou	2007	50	other	3	S	S	–
Le Wang	2019	53	16S	6	C	NS	K–B
Jing Wang	2018	57	other	13	C	NS	–
Jin Li	2014	58	other	3	C	NS	–
Wei Liu	2006	60	other	2	–	NS	–
Dongyang He	2006	64	other	2	CS	S	–
Ning Zhu	2020	71	other	8	–	S	MIC
Xurong Wang	2012	76	other	3	–	NS	–
Zhiyuan Wang	2002	85	other	1	CS	NS	–
Haiping Deng	2007	100	other	2	–	NS	–
Yonghua Qi	2006	102	other	3	–	NS	–
Huiyun Zhao	2020	110	16S	2	C	NS	–
Guiying Wang	2008	115	other	3	C	NS	–
Jidong Zhang	2006	150	other	9	CS	–	–
Lijun Wu	2019	165	16S	2	S	S	–
Jie Tan	2014	166	other	5	CS	NS	–
Xiaohui Feng	2019	200	16S	32	–	NS	MIC
Yingying Ge	2019	210	other	5	–	NS	–
Airi Ha	2018	212	other	55	–	NS	–
Javed Memon	2012	217	16S	11	S	NS	–
Yuxiang Shi	2020	245	16S	45	C	NS	K–B
Huarong Song	2009	260	other	6	S	NS	–
Hongsheng Li	2002	280	16S	4	–	NS	–
Xinpu Li	2015	302	16S	1	C	NS	–
Bo Yang	2009	370	16S	4	S	NS	–
Jia Cheng	2020	916	16S	206	CS	NS	MIC
Zhe Zhang	2019	1122	16S	18	–	–	–
Xiangbin Song	2020	1153	16S	23	CS	NS	–
Limei Wang	2007	1456	other	13	CS	NS	–
Sanping Bo	2014	1716	other	78	–	NS	–
Jian Gao	2017	3190	16S	426	C	NS	–

*1.16S means 16S rDNA sequencing. 2. C, clinical bovine mastitis; S, subclinical bovine mastitis; CS, clinical and subclinical bovine mastitis. 3. S, South China; N, North China; NS, North and South China. 4. K–B, disk diffusion, MIC, broth microdilution. “–” means the information was not declared in the original articles*.

### Statistical Analysis

Data were extracted from individual studies using a predesigned form obtaining data on the author, year, province, the number of samples, the number of Klebsiella isolates, mastitis grade (clinical and subclinical mastitis criterion: Laboratory handbook on bovine mastitis and National Mastitis Council), bacterial identification methods, the number of antibiotic-resistant isolates, and laboratory procedures. The same two reviewers independently, and in duplicate, assessed the methodological quality of each individual study based on the prespecified study quality indicators adapted from the Downs and Black checklist.

The numbers of *Klebsiella* spp., antimicrobial-resistant isolates, and mastitis milk samples within individual studies were calculated for their proportion. Resistance was considered a dichotomous outcome, as classified by individual primary studies. Isolates with intermediate susceptibility were classified as susceptible.

Meta-analyses were performed separately for *Klebsiella* spp. prevalence and their AMR rates. This procedure was performed by using the “meta” and “metafor” package in R (Version 4.0.5) and only conducted if four or more studies were considered because between-study variance cannot be estimated accurately when it is less than this number and may result in biased pooled estimates after the meta-analysis.

We pooled the prevalence of *Klebsiella* spp. using random effects models. Subgroup meta-analyses were conducted for isolation time, isolate regions, and mastitis grade to illustrate the heterogeneity between the included studies.

For the AMR studies, we pooled analyses within nine groups: β-lactams, quinolones, aminoglycosides, tetracyclines, polypeptides, sulfonamides, amphenicol, macrolides, and lincosamides.

A publication bias test was performed by using “Egger” test, and the funnel plot was created. Sensitivity analysis was conducted by using “leave-one-out” analyses. Both of them were conducted by using the “meta” and “metafor” packages in R (version 4.0.5).

## Results

### Inclusion of Publications

A total of 34, 86, and 166 articles were obtained from PubMed, Google scholar, and CNKI, respectively. Among them, 24 publications were duplicated, a total of 31 publications were excluded because they did not declare the sample size nor the number of bacterial isolates, and two publications used a small sample size, which were excluded. In addition, 16 articles contained non-mastitis cases, 159 publications did not contain *Klebsiella* spp. cases, 11 articles were beyond the considered period (before 2000), four articles were reviews, and one article did not declare the identification method. Therefore, these publications were denied. Finally, a total of 38 full-textt publications were included in our research, of which 7 covered the AMR test (Figure 1).

As for the 38 publications, two publications did not describe the sample collected location exactly, they were included and given our focus on the prevalence of Klebsiella throughout the country. A total of seven publications obtained clinical and subclinical samples, and 14 did not describe the grade of mastitis. However, we still included them in our research because we focused on the whole condition of bovine mastitis (Table 1).

### Prevalence of *Klebsiella* spp.

**Figure 2:**
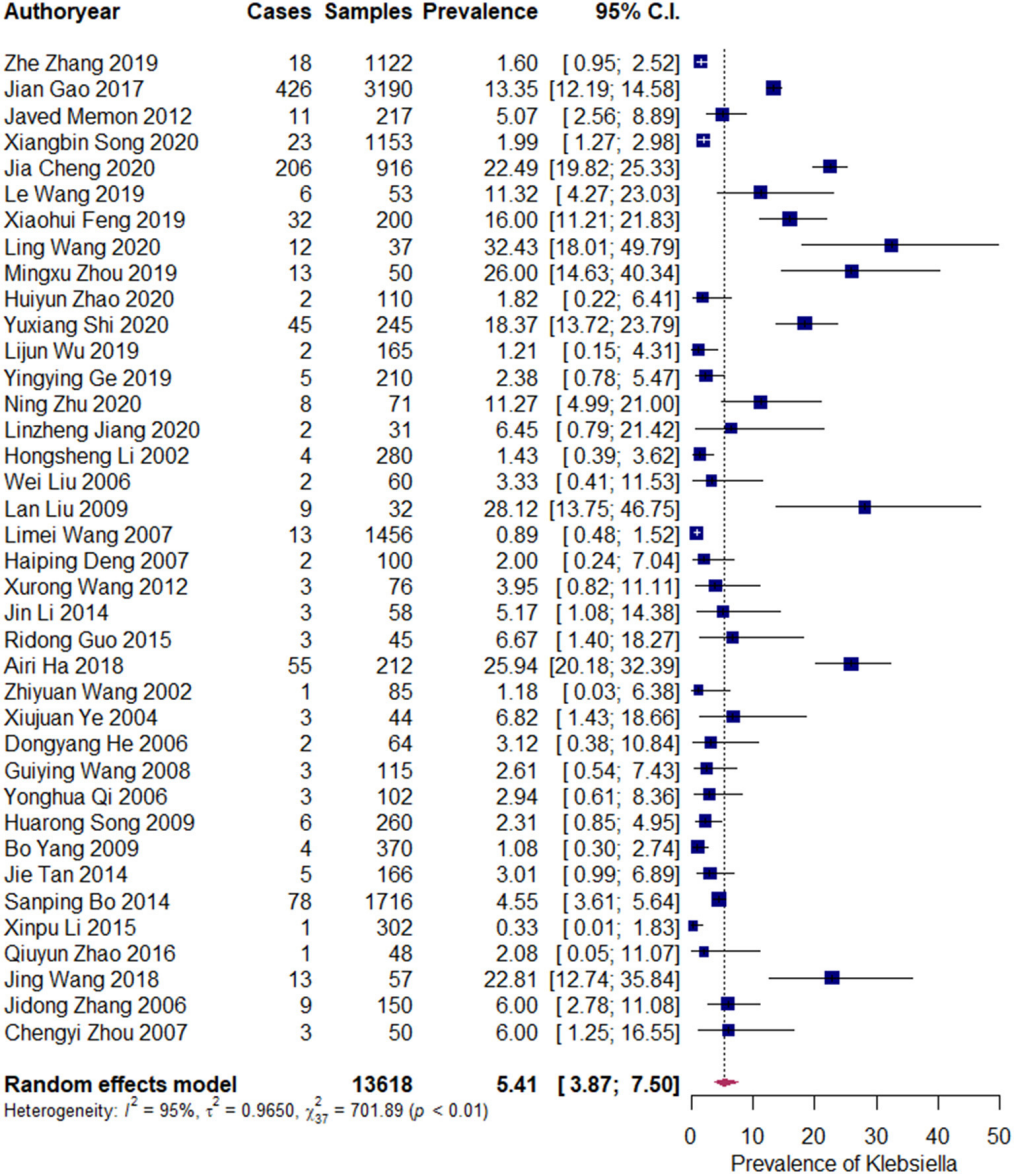
Prevalence of *Klebsiella* spp. Each navy blue square represents the mean prevalence for a study. The upper and lower limit of the line connected to the square represents the upper and lower 95% CI for the prevalence. The size of the square reflects the relative weighting of the study to the overall prevalence estimate, with larger squares representing greater weight. The broken vertical line represents overall prevalence and the diamond at the bottom represents the overall prevalence (location of the diamond) and its 95% CI (width of the diamond). Cases refers to the *Klebsiella* spp. containing samples. “Author year” refers to the first author and publication year of the publication (the same below).

The pooled prevalence of *Klebsiella* spp. was 5.41% (95% CI: 3.87–7.50%). An evident heterogeneity was observed (*I*^2 =^ 95%, τ^2^ = 0.965, *P* <0.01). Therefore, a subgroup analysis was conducted to explore the sources of heterogeneity (Figure 2).

### Subgroup Analysis

We divided the research articles into subgroups based on the research period (2000–2010 vs. 2010–2020), sample sites (North China vs. South China), and mastitis grade (clinical mastitis vs. subclinical mastitis). The pooled prevalence values of *Klebsiella* spp. were 3.14 and 7.45% (2000–2010 vs. 2010–2020, Figure 3); 7.49 and 4.03% (clinical mastitis vs. subclinical mastitis, Figure 4); 4.22 and 8.55% (North China vs. South China, Figure 5), respectively.

**Figure 3:**
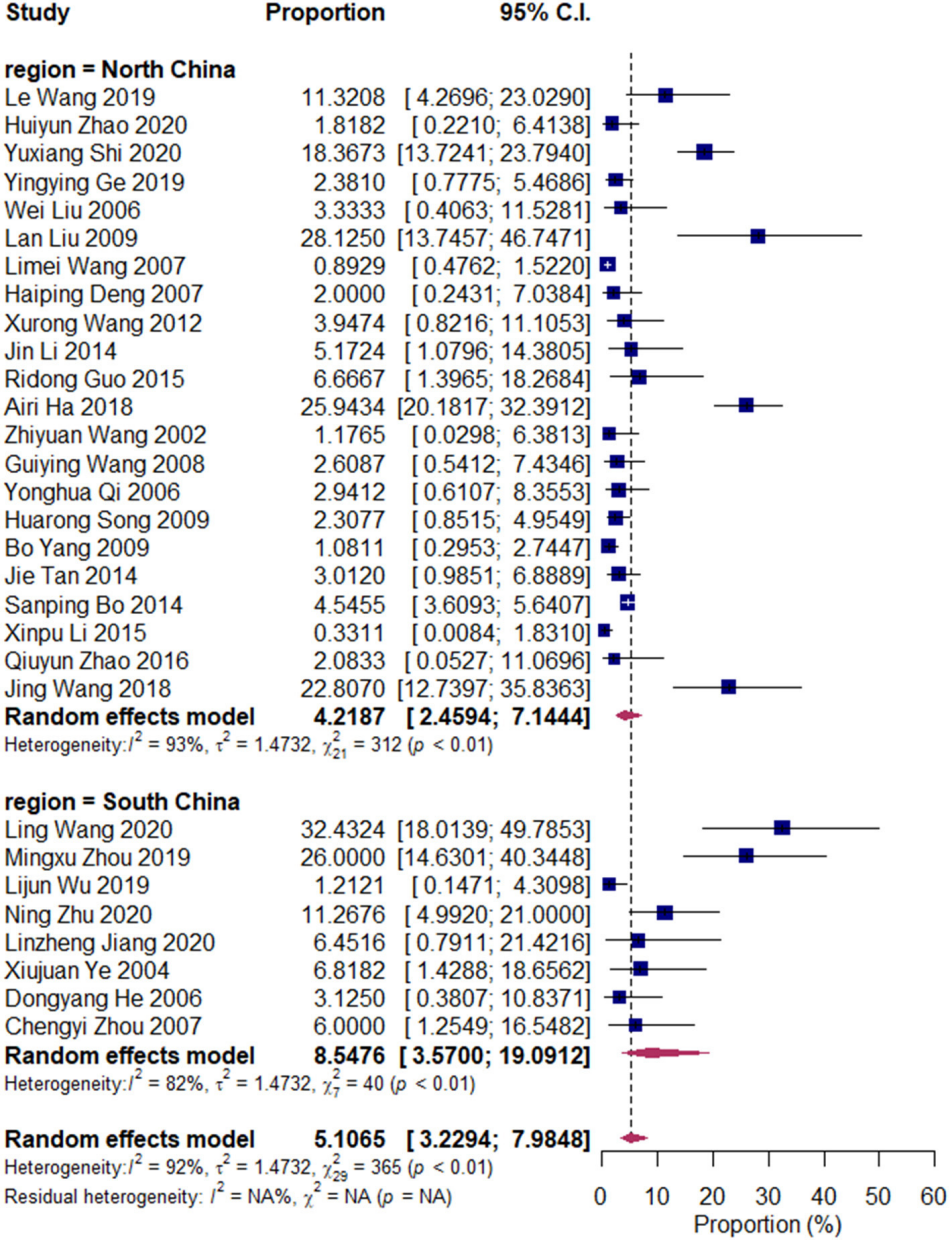
Prevalence of *Klebsiella* spp. in North and South China. Subgroups were created according to the sampling region in China (North China/South China). The two upper diamonds represent the overall prevalence within the respective subgroups, whereas the diamond at the bottom represents the overall prevalence of all studies (the same below).

**Figure 4:**
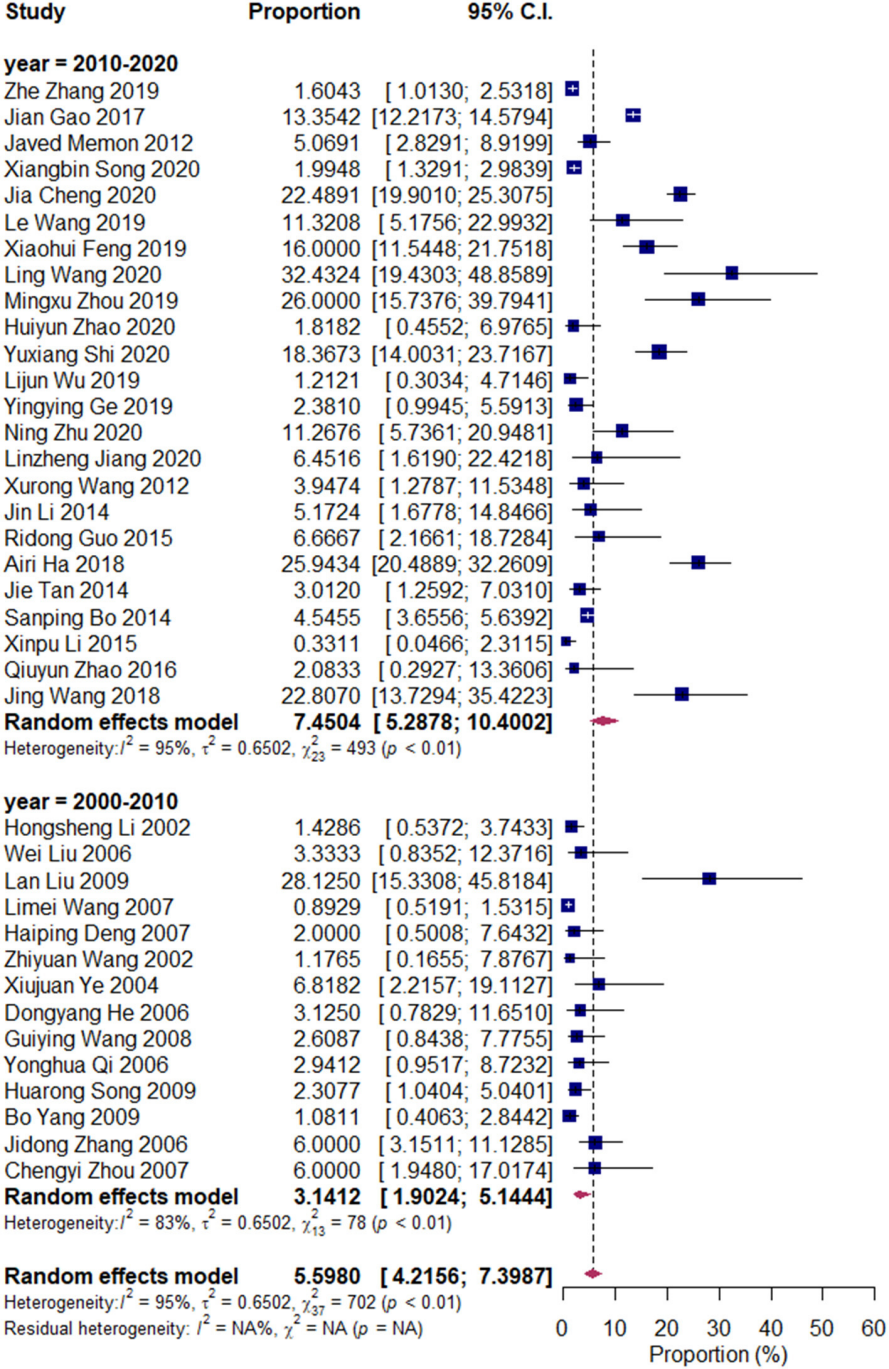
Prevalence of *Klebsiella* spp. in the period of 2000–2010 and 2010–2020. Subgroups were created according to the publication year (2000–2010/2010–2020).

**Figure 5:**
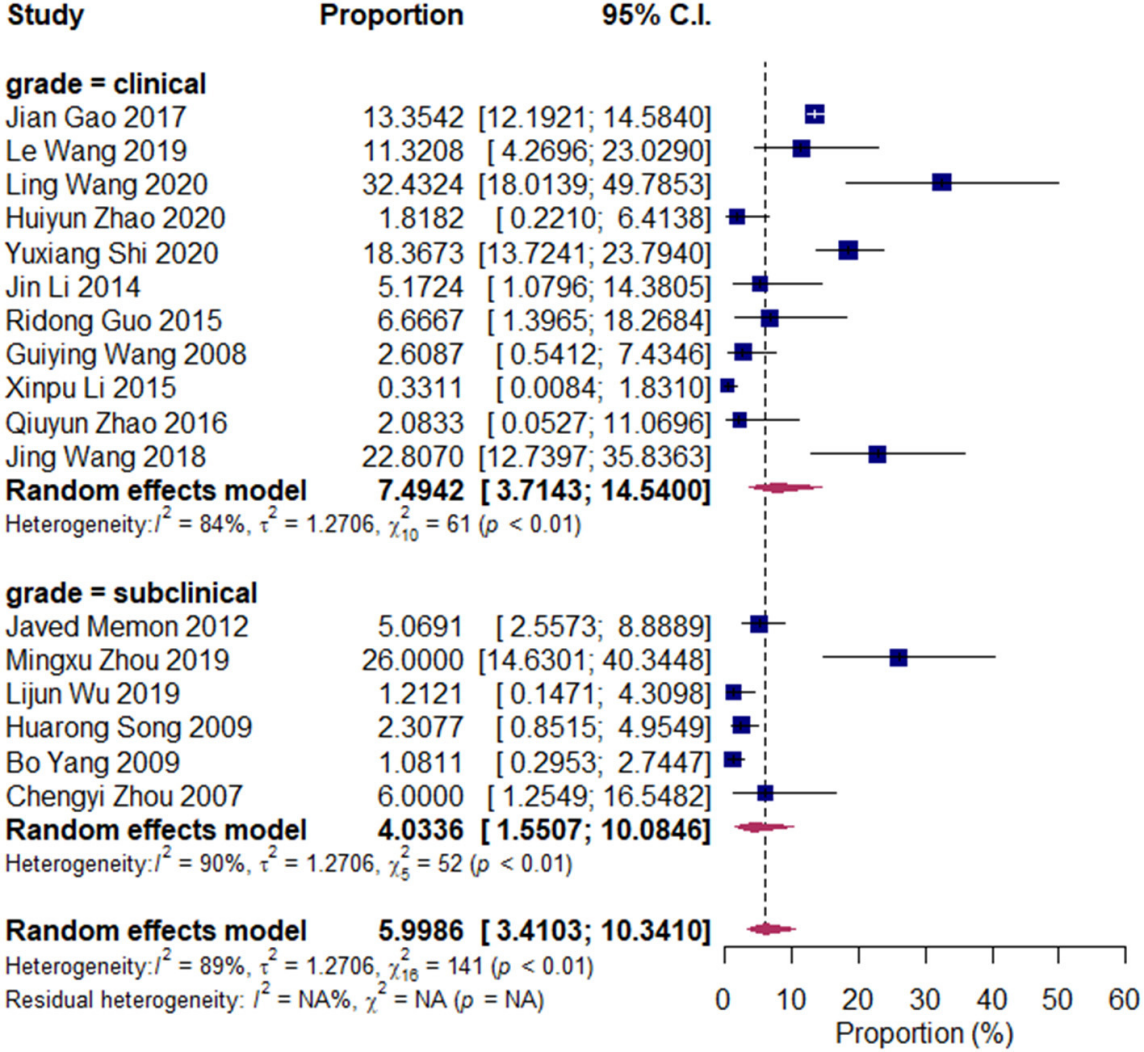
Prevalence of *Klebsiella* spp. isolated in clinical mastitis and subclinical mastitis cases. Subgroups were created according to the bovine mastitis grade (clinical/subclinical).

### Publication Bias of the Prevalence of *Klebsiella* spp.

**Figure 6:**
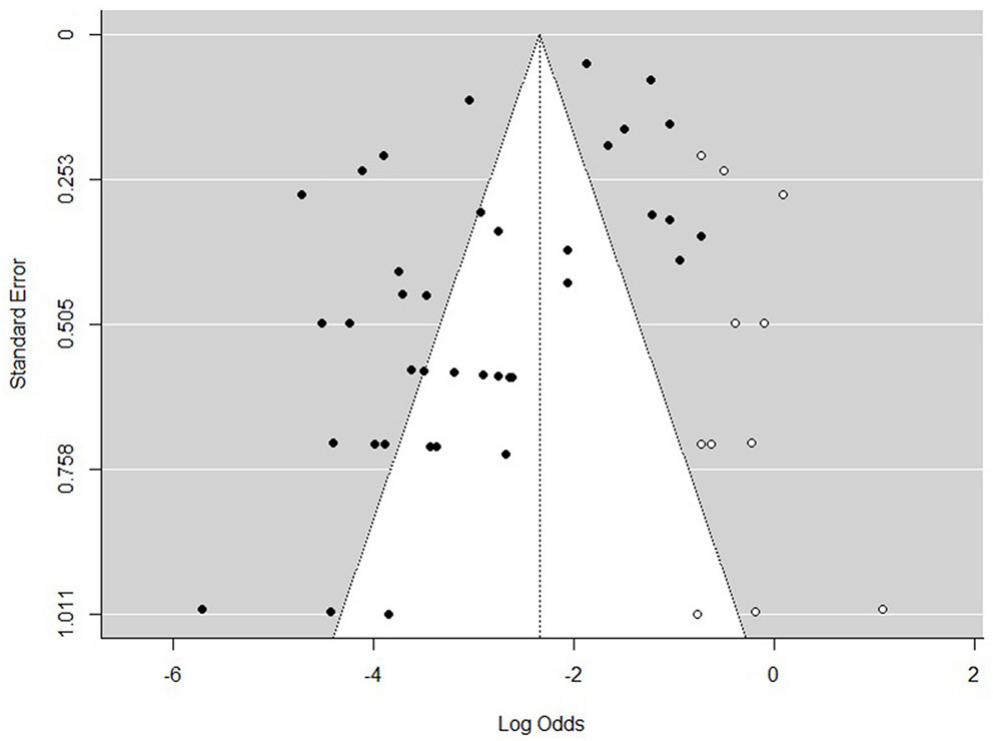
Funnel plot of publication bias of prevalence of *Klebsiella* spp.

The funnel plot (Figure 6) exhibited an even distribution of the studies around the mean effect size, which suggested that the publication bias was not evident.

### Antimicrobial Resistant Rate of *Klebsiella* spp.

**Figure 7:**
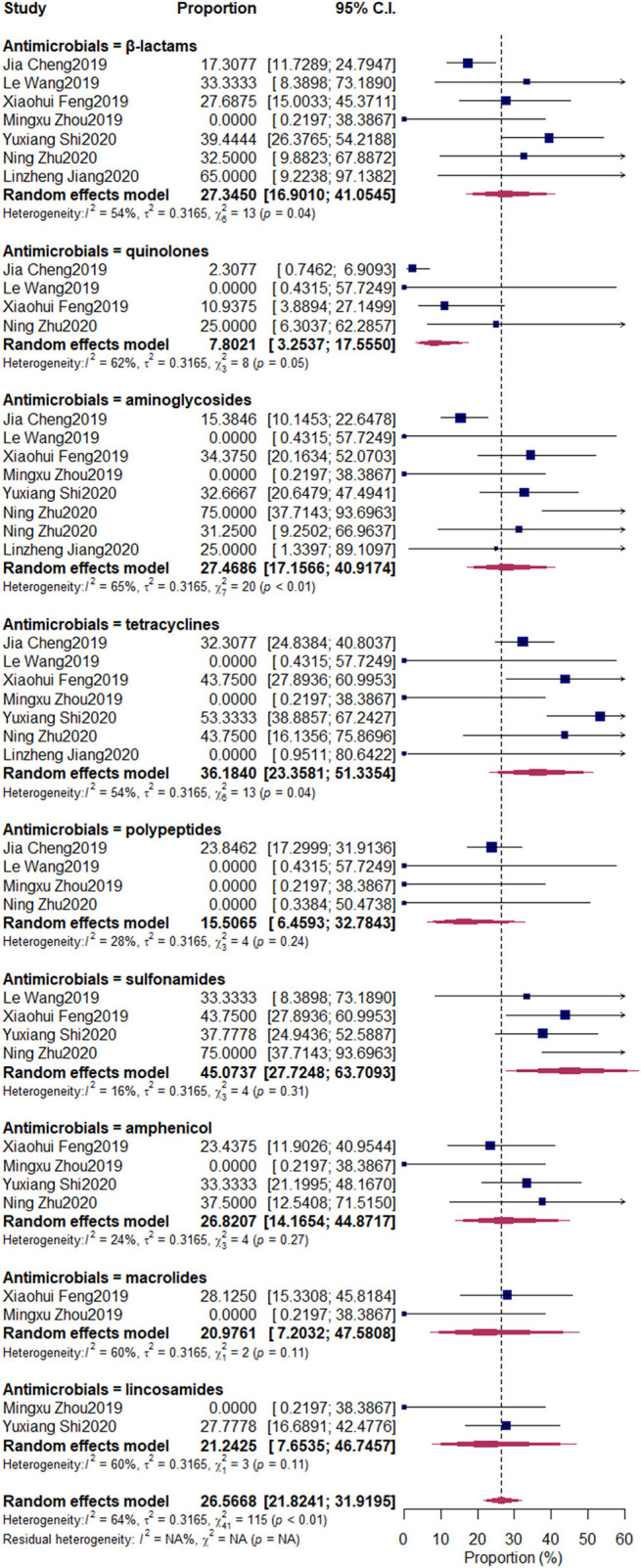
Antimicrobial-resistant rate of *Klebsiella* spp. Subgroups were created according to the different kinds of antimicrobials. The nine upper diamonds represent the overall antimicrobial resistance (AMR) rate within the respective subgroups, whereas the diamond at the bottom represents the overall AMR rate of all studies.

The pooled resistant rates were as follows: β-lactams, 27.35% (95% CI: 11.73–24.79%); quinolones, 7.8% (95% CI: 3.25–17.56%); aminoglycosides, 27.47% (95% CI: 17.16–40.92%); tetracyclines, 36.18% (95% CI: 23.36–51.34%); polypeptides, 15.51% (95% CI: 6.46–32.78%); sulfonamides, 45.07% (95% CI: 27.72–63.71%); amphenicol, 26.82% (95% CI: 14.17–44.87%); macrolides, 20.98% (95% CI: 7.20–47.58%); lincosamides, 22.24% (95% CI: 7.65–46.75%) (Figure 7).

### Publication Bias of the AMR Rate of *Klebsiella* spp.

**Figure 8:**
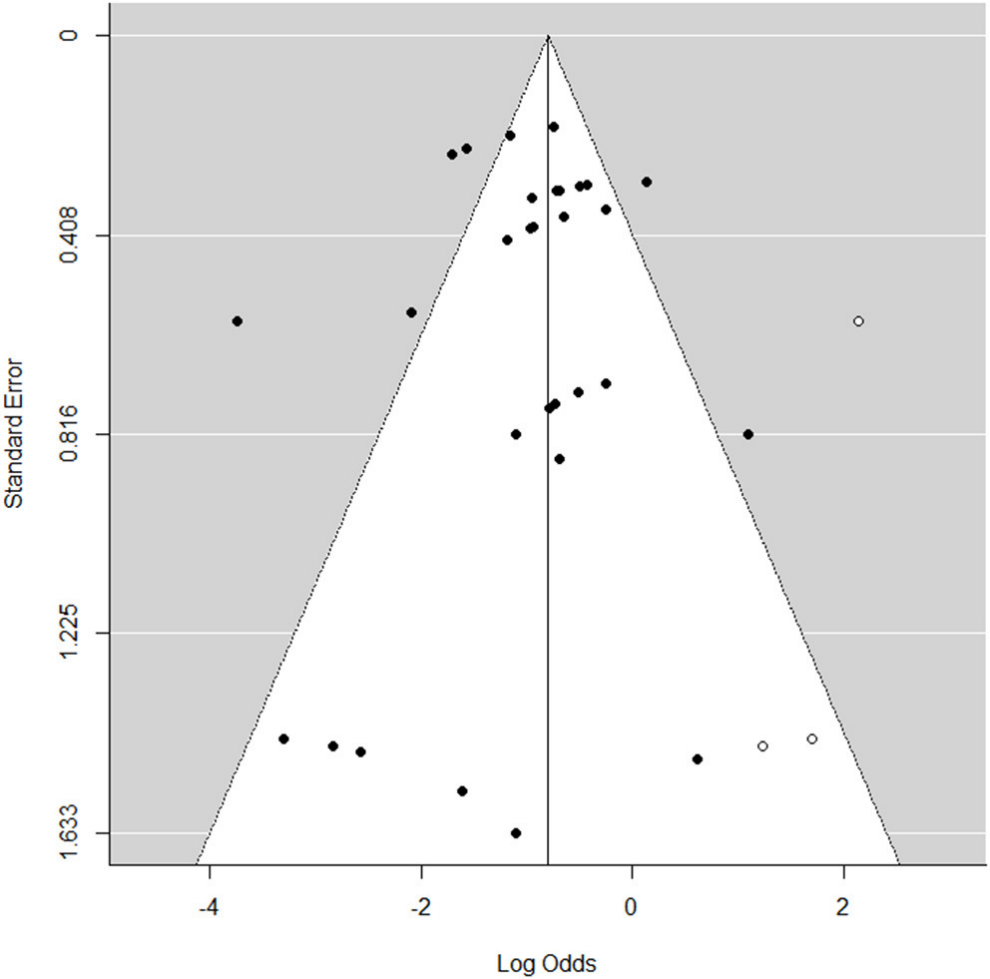
Funnel plot publication bias of antimicrobial resistance rate of *Klebsiella* spp.

The funnel plot (Figure 8) exhibited an even distribution of the studies around the mean effect size, which suggested a negligible publication bias.

## Discussions

Bovine mastitis is the costliest disease in the dairy industry (26). *Klebsiella* spp. is important pathogens causing bovine mastitis and human infection (27, 28). Understanding the prevalence and AMR profiling of bovine mastitis, *Klebsiella* spp. may contribute to therapeutic interventions and preventive strategies.

A total of 38 publications, 13,618 samples, and 1,037 isolates were pooled in our study. The pooled prevalence of *Klebsiella* spp. was 5.41% (95% CI: 3.87–7.50%). Subgroup meta-analysis indicated that the prevalence of *Klebsiella* spp. in South China was higher than that in North China, that in subclinical mastitis was lower than that in clinical mastitis, and that in 2000–2010 was lower than that in 2010–2020.

Our results revealed that the pooled prevalence of *Klebsiella* spp. was relatively lower than those of previous studies conducted in China (13 and 9.78%) (9, 29). Meanwhile, the prevalence of *Klebsiella* spp. in South China (8.5%) was twice that in North China (4.2%). Environmental sources, such as alleyways, holding pens, and sawdust and shavings in bedding, are important sources of *Klebsiella* spp. (National Mastitis Council, 1999; (28, 30). Our results were consistent with those of Gao et al., whose results have revealed that the prevalence of *Klebsiella* spp. in Northwest China is lower than that in South China, and that in winter was lower than that in summer; this finding is attributed to the dry and cold weather in North China, which is unsuitable for environmental microorganisms (9).

In recent years, *Klebsiella* spp. mastitis, which is attributed to the fecal shedding of *Klebsiella* spp., increased the concern for herds that use inorganic bedding (31). *Klebsiella pneumoniae* is an endophyte of several plants, such as wheat, corn, and alfalfa, and it can act as milk cow feed; bacteria can be found inside the plants without external fecal contamination (32–34). Consequently, the oral intake of *Klebsiella* spp. can be due to the plants used for feed or to fecal contamination of feed and water, whereas fecal shedding of *Klebsiella* spp. results in the contamination of the environment of cows. Such sources provide dairy herd managers and veterinarians with additional control points for the prevention of *Klebsiella* spp. mastitis. The increased prevalence of *Klebsiella* from 2000 to 2020 raised the concern for this important mastitis pathogen.

Cheng et al. (35) revealed that *Klebsiella* spp. can induce severe and long-term infection in the bovine milk gland, and can give rise to clinical bovine mastitis more than the subclinical version. Our results also revealed that the prevalence of *Klebsiella* spp. in clinical mastitis is higher than that in subclinical bovine mastitis.

*Klebsiella* spp. can induce bovine mastitis; antimicrobial treatment is normally used for mastitis prevention and control (7).

The misuse of antimicrobials can increase the risk of AMR and threaten public health. (36). In our research, the AMR of *Klebsiella* spp. against nine kinds of frequently used antimicrobials (β-lactams, quinolones, aminoglycosides, tetracyclines, polypeptides, sulfonamides, amphenicol, macrolides, and lincosamides) was determined. We first pooled nationwide studies that were conducted to determine the AMR of *Klebsiella* spp. isolated from bovine mastitis in China. For these nine antimicrobials, the resistance rate of sulfonamides was the highest (45.07%), followed by tetracyclines (36.18%), aminoglycosides (27.47%), β-lactams (27.34%), amphenicol (26.82%), lincosamides (21.24%), macrolides (20.98%), polypeptides (15.51%), and quinolones (7.80%).

In a previous study conducted by Saini et al. (36), the resistance to sulfonamides (11.7%) and β-lactams (17.3%) was the highest, but the values were still lower than that in our research. In another study conducted in Canada, *Klebsiella* spp. was the main pathogen resistant to tetracyclines (19%) and streptomycin (38%) (37). The high percentages of AMR of *Klebsiella* spp. against sulfonamides, β-lactams, amphenicol, lincosamides, macrolides, and polypeptides in that study were not observed, similar to the observations in European countries (38), in which the resistance to streptomycin was higher than that in our research; however, the resistance to tetracyclines was lower than that in our study. The use of sulfonamides and tetracyclines in husbandries had been forbidden by the Chinese government. However, the observed AMR rate was still high, which indicated that the AMR mechanism was still harbored by *Klebsiella* spp. aminoglycosides and β-lactams should raise the most concern when used in treating bovine mastitis. Tetracyclines are one of the extensively used antimicrobials among dairy farms. Its AMR has been a serious problem before, but with the increased awareness of public health in society, the Chinese government imposed a ban on the use of antimicrobials as growth promoters in the husbandry industry, which restricted the AMR of pathogens.

Fuenzalida and Ruegg (39) indicated that the cure rate for *Klebsiella pneumoniae* mastitis was 21% greater in 8-day than in 2-day intramammary ceftiofur group. A previous study conducted in China by Cheng et al. (35) reported that *Klebsiella* spp. were also highly resistant to amoxiclav (38%), with a value higher than that in the study of Schukken et al. (11) in the USA. Our results also indicated that the AMR of bovine mastitis-associated *Klebsiella* spp. against β-lactams in China was as high as 27.34%. Yang et al. (40) reported that the β-lactam resistance gene *bla*CTM-M-1 located on pC5-like plasmids can be responsible for the resistance against ceftiofur for bovine mastitis treatment. Schukken et al. (11) suggested that the antimicrobial treatment of *Klebsiella* spp. bovine mastitis has a minimal value, and heteropathy for clinical symptoms should be the primary goal. A recent study indicated that third-generation cephalosporin and carbapenems will be ineffective against a large proportion of *Klebsiella* spp. in most parts of the world by 2030 (19), which should raise the concern for the AMR of *Klebsiella* spp. associated with bovine mastitis.

The results in our study were consistent with those of Cheng et al. (35), whose results indicated that the AMR occurrence rates of five common bovine mastitis pathogens, including *Klebsiella* spp., in China were higher than those in the European countries (41). The occurrence rate of AMR among bovine mastitis pathogens differs among various countries (42), and this condition can be attributed to complex reasons, such as the national guidelines for proper antibiotics usage, veterinarian prescription patterns, and pharmaceutical marketing strategies (43, 44). Hence, our results should raise the concern about the AMR of bovine mastitis Klebsiella spp. in Chinese dairy herds. There are still limitations including few databases retrieval and publication time substituting sample time in our manuscript, which should make improvements in the future.

## Conclusions

The pooled prevalence of *Klebsiella* spp. was 5.41% (95% CI: 3.87–7.50%). Subgroup analysis revealed that the incidence was higher in South China, from 2010 to 2020, and in clinical bovine mastitis cases, and the reason is attributed to the climate between South and North China and the natural pathogenicity of *Klebsiella* spp. The pooled AMR rates showed that *Klebsiella* spp. were most resistant to sulfonamides, followed by tetracyclines, aminoglycosides, β-lactams, amphenicol, lincosamides, macrolides, polypeptides, and quinolones, which should raise the most concern when used in treating bovine mastitis.

## Data Availability Statement

The original contributions presented in the study are included in the article/supplementary material, further inquiries can be directed to the corresponding author/s.

## Author Contributions

KL designed the study, analyzed the data, and wrote the article. LZ and XG performed the literature research and review. WQ critically reviewed and revised the manuscript. All authors contributed to the article and approved the submitted version.

## Conflict of Interest

The authors declare that the research was conducted in the absence of any commercial or financial relationships that could be construed as a potential conflict of interest.

## Publisher's Note

All claims expressed in this article are solely those of the authors and do not necessarily represent those of their affiliated organizations, or those of the publisher, the editors and the reviewers. Any product that may be evaluated in this article, or claim that may be made by its manufacturer, is not guaranteed or endorsed by the publisher.
